# Regression of the Left Ventricular Hypertrophy in Patients with Essential Hypertension on Standard Drug Therapy

**DOI:** 10.15190/d.2020.12

**Published:** 2020-09-30

**Authors:** Shah Newaz Ahmed, Ratinder Jhaj, Balakrishnan Sadasivam, Rajnish Joshi

**Affiliations:** ^1^Department of Pharmacology, All India Institute of Medical Sciences Bhopal, Madhya Pradesh, India; ^2^Department of General Medicine, All India Institute of Medical Sciences Bhopal, Madhya Pradesh, India

**Keywords:** Hypertension, left ventricular hypertrophy, regression, thiazides, calcium channel blockers

## Abstract

PURPOSE: The American College of Cardiology/ American Heart Association 2017 and European Society of Cardiology/European Society of Hypertension 2018 guidelines were a paradigm shift in hypertension management in contemporary medicine. Lowering of blood pressure to less than 130 (systolic) and 80 (diastolic) mm of Hg irrespective of cardiovascular risk is recommended. While intensive blood pressure control is commonly achievable with rational pharmacotherapy, the magnitude of left ventricular hypertrophy regression is an independent factor in improvement in cardiovascular health. The regression of left ventricular hypertrophy has been adjudged as a clinically useful surrogate marker that reflects the efficacy of hypertension treatment. Though angiotensin converting enzyme inhibitors/ angiotensin receptor blockers (ACEI/ARB) are the preferred initial drug for greater regression of left ventricular mass, the choice of add-on therapy, if required, is still debatable. Therefore, in our observational study, we sought to compare the reduction in left ventricular mass index in hypertensives with left ventricular hypertrophy on standard ACEI/ARB based drug therapy. MATERIALS AND METHODS: The cohort (n=217) comprised of patients with uncontrolled hypertension (blood pressure>140/90 mm of Hg) and left ventricular hypertrophy (left ventricular mass index>115 and 95 gram/square meter in males and females respectively). The add-on drug in ACEI/ARB therapy was either thiazide diuretics (TD) or calcium channel blockers (CCB). Four sub-cohorts were constituted: mono-therapy - group A (n=70, ACEI/ARB), dual-therapy - group B (n=48, ACEI/ARB+TD) and  group C (n=51, ACEI/ ARB+CCB), triple therapy - group D (n=48, ACEI/ ARB+TD+CCB). Left ventricular mass index was determined using echocardiography at baseline and after 24 weeks of therapy. RESULTS: There was no significant difference in baseline clinical or demographic variables between group B and group C. Baseline blood pressure and duration of hypertension was greater in group D compared to group A (P<0.001). The reduction in left ventricular mass index (mean ±SD) in the four groups (A to D) was 16.7±18.7, 21.0±20.8, 20.5±15.5  and 29.1±21.5 g/m2  respectively (D>A, P=0.011, B versus C, P=1.00). The corresponding change in blood pressure (systolic/diastolic) was 18.5±13.6/8.9±11.2, 27.5±19.2/12.2±9.3, 23.4±16.7/ 5.4±10.1, 26.6±19.5/10.7±12.8 mm of Hg respectively (systolic, B>A, P=0.027, D>A, P=0.048) (diastolic, B>C, P=0.013). CONCLUSION: Anti-hypertensive treatment with angiotensin converting enzyme inhibitors/angiotensin receptor blockers-based therapy produced graded regression of left ventricular hypertrophy with monotherapy, dual therapy and triple therapy.  In dual therapy, add-on of either thiazide diuretics or calcium channel blockers to angiotensin converting enzyme inhibitors/angiotensin receptor blockers showed equal efficacy in regression of left ventricular hypertrophy independent of blood pressure reduction.

## INTRODUCTION

The left ventricle of the heart shows a time-dependent, compensatory response to blood pressure (BP) resulting in adaptive change in myocardial thickness. In patients of primary hypertension, increase in left ventricular mass occurs in response to chronic pressure overload that tends to stabilize the equilibrium between the left ventricular wall stress and the increased BP. An exaggerated increase in myocardial thickness in due course of time leads to concentric left ventricular hypertrophy (LVH)^1^. LVH is prevalent in 36 to 41% of patients with essential hypertension^2,3^. A definite association has been established, not only between hypertension and LVH, but also between LVH and cardiovascular events, both of which, cumulatively and independently, increase the risk of cardiovascular mortality and morbidity^4^. LVH is independently associated with four-fold greater risk of cardiovascular events^4-9^. Statistically, a mathematical relationship exists between left ventricular mass in grams and the rate of cardiovascular events^4^. However, pharmaco-therapy directed at reducing elevated BP reduces complications associated with hypertension. It has also been observed that with prolonged control of BP there is regression of LVH with significant decrease of unfavourable clinical outcomes. There are plenty of studies that show the attenuation of the risk of adverse cardiovascular events with regression of LVH^5-9^. Therefore, regression of LVH has been adjudged as a clinically useful surrogate marker that reflects the efficacy of hypertension treatment^10^.In patients of hypertension with diabetes, it has been shown that greater lowering of BP leads to greater reduction in the risk of LVH^11^. Angiotensin converting enzyme inhibitors/angiotensin receptor blockers (ACEIs/ARBs), unless otherwise contraindicated, are drugs of first choice in patients with essential hypertension with LVH^12-16^. Most patients require a second drug in the long run for optimum control of BP. Current guidelines recommend treatment of hypertension with a combination of two drugs for BP above 140/90 (American College of Cardiology/American Heart Association 2017 (ACC/AHA2017) guidelines), or 160/100 (Joint National Committee 7/8 (JNC7/8) guidelines) or 150/90 (European Society of Cardiology/European Society of Hypertension (ESC/ESH2018) guidelines). While ACC and JNC guidelines allow use of combination of any two of the first line drugs, the ESC/ESH 2018 guidelines recommend preferred initial combination of ACEIs/ARBs with calcium channel blockers (CCB) or diuretics. In absence of a compelling indication, it is left to the physician’s discretion to decide upon the add-on therapy^17-19^. In our work, we studied the efficacy of ACEIs/ARBs based regimen as monotherapy or as combination with CCB and thiazide diuretics (TD) in regression of hypertensive LVH.

## MATERIALS AND METHODS

### Study design and ethical approval

An observational prospective (cohort) study was conducted to compare the regression of LVH in patients with essential hypertension attending the medicine out-patient department at AIIMS, Bhopal. The study was conducted with the ethical approval of the Institutional Human Ethics Committee, AIIMS, Bhopal. All procedures performed in studies involving human participants were in accordance with the ethical standards of the institutional and/or national research committee and with the 1964 Helsinki declaration and its later amendments or comparable ethical standards. Informed consent was obtained from all individual participants included in the study.

### Sample size

The sample size was calculated using effect size. Effect size is a standard measure of the difference between two or more groups independent of the unit of the outcome variable. For simplicity, effect size is categorised into three levels-small, medium and large. Keeping alpha probability of 0.05 and power of 0.80, in a one-way ANOVA with fixed effects single factor design, the calculated values of the effect size at small, medium and large levels are 0.1, 0.25 and 0.4 respectively^20^. Based on clinical judgement and practical feasibility, we decided to use a medium effect size in our study. The total sample size for the specified effect size was calculated as 180 for four independent groups. Thus, 45 patients were required per group for the study. The sample size estimation was carried out using G*power version 3.1.9.2. Taking into account that 20-40% of patients may be lost to follow up, we decided to recruit 300 patients in our study.

### Selection of study participants

All participants in the study were patients of uncontrolled essential hypertension. The inclusion criteria for the study were: 1. Age between 35 and 85 years old; 2. Blood pressure > 140/90 mm of Hg; 3. Concentric left ventricular hypertrophy left ventricular mass index (LVMI)>115 g/m^2^ in men and>95 g/m^2^ in women). The exclusion criteria were: 1. Chronic kidney disease (GFR < 60ml/min/1.73 m^2^); 2. Pregnancy and women in childbearing age group not on contraceptives; 3. Congestive cardiac failure New York Heart Association (NYHA) class II-IV; 4. Valvular heart disease, cardiac arrhythmia, 2nd or 3rd degree heart block, sick sinus syndrome; 5. Post myocardial infarction with regional wall motion abnormality or ejection fraction < 50%; 6. Known bilateral renal artery stenosis; 7. Secondary hypertension; 8. Chronic liver disease (AST/ALT values>3 times upper limit of normal).

The study compared the efficacy of different regimens of antihypertensive drugs in regression of LVH. This comparative analysis did not disturb the treatment protocol, which was delivered according to existing standards of care. Patients with uncontrolled hypertension who had LVH based on echocardiography were recruited in one of the four groups (group A (ACEIs/ARBs), group B (ACEIs/ARBs+CCB), group C (ACEIs/ARBs+TD), group D (ACEIs/ARBs+CCB+TD))

### Outcome measures

#### Primary outcome measure:

· Change in LVMI following continuous treatment under one of the drug regimens for a period of six months.

#### Secondary outcome measure:

· Change in blood pressure during the same period.

### Method of determination of LVM and LVMI by echocardiography

**Figure 1:**
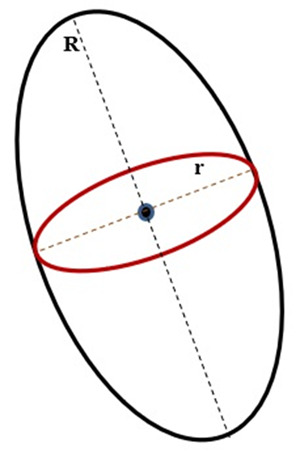
Shape of the left ventricle is analogous to a prolate ellipse A prolate ellipse is a surface of revolution obtained by rotating an ellipse about its major axis. R and r are major and minor axis respectively.

The shape of the left ventricle of the heart is analogous to a prolate ellipse (Figure 1).

Mathematically, the volume of a prolate ellipse can be determined as:

V=4/3(πr^2^R)

where V is volume, R is major axis and r is minor axis.

The length of the major axis of the left ventricle is twice the minor axis,

such that when r = R/2,

V=(4/3)π(R/2)^2^R

or, V=1.05R^3^

The left ventricular internal and total volumes are calculated on the basis of this formula:

· Left ventricular internal cavity volume (V _I_) = 1.05 (left ventricular internal dimension)^3^

· Total left ventricular volume (V_ T_) = 1.05 [(inter ventricular septum thickness (IVST) + left ventricular internal dimension (LVID) + posterior wall thickness (PWT)]^3^

Left ventricular myocardial volume (V _M_) = V_T_ - V_I_

Left ventricular mass (LVM)= V_M _X myocardial density.

All measurements were taken according to the recommendations of the American Society of Echocardiography (ASE). After correction for factors derived from regression analysis, the formula for determination of LVM is:

LVM=0.8[1.05(LVID+IVST+PWT)^3^–(LVID)^3^]+0.6^21^.

LVMI=LVM/Body surface area

Body surface area= 0.007184 x W^0.425^ x H^0.725^ ^22^

(W= weight, H= height)

As per the latest ASE guidelines, for designating a case as echocardiography detected LVH, LVMI should be more than 115 g/m^2^ and 95 g/m^2 ^for men and women respectively. Alternatively, inter-ventricular septal wall thickness (IVST) should be more than 1.0 cm and 0.9 cm in men and women respectively^7^.

### Measurement of office blood pressure

Blood pressure was recorded with a properly calibrated aneroid sphygmomanometer in the dominant arm. The patient was made to comfortably seat in a chair for 5 minutes with feet on the ground. He/she had no physical activity at least 30 minutes prior to record of blood pressure. The elbow was extended, and the instrument was positioned at the level of heart. The arm was bared and snugly wrapped with the cuff 2 fingers above the elbow crease covering more than 2/3^rd^ of the arm circumference. At first the systolic pressure was determined by the palpatory method, and then both the systolic and diastolic pressure were determined by the auscultatory method. Two such readings were taken two minutes apart and the mean of the two readings was recorded.

### Statistical analysis

The data collected in this study was analysed using SPSS version 22.0. The data were expressed as mean ± SD, or as count and percentage. Baseline differences in categorical variables between the study groups were examined by Pearson’s chi-square or Fisher’s exact tests. Baseline differences in continuous variables between the study groups was examined by analysis of variance (ANOVA) test. Analysis of Covariance (ANCOVA) was applied to find presence of significant difference in primary outcome measure between the treatment arms at the completion of the study. Post hoc Bonferroni test or Tukey’s HSD was used to find out intergroup differences. P value <0.05 was considered statistically significant. Significant changes in blood pressure and LVMI before and after treatment was analysed using paired t-test.

## RESULTS

**Figure 2:**
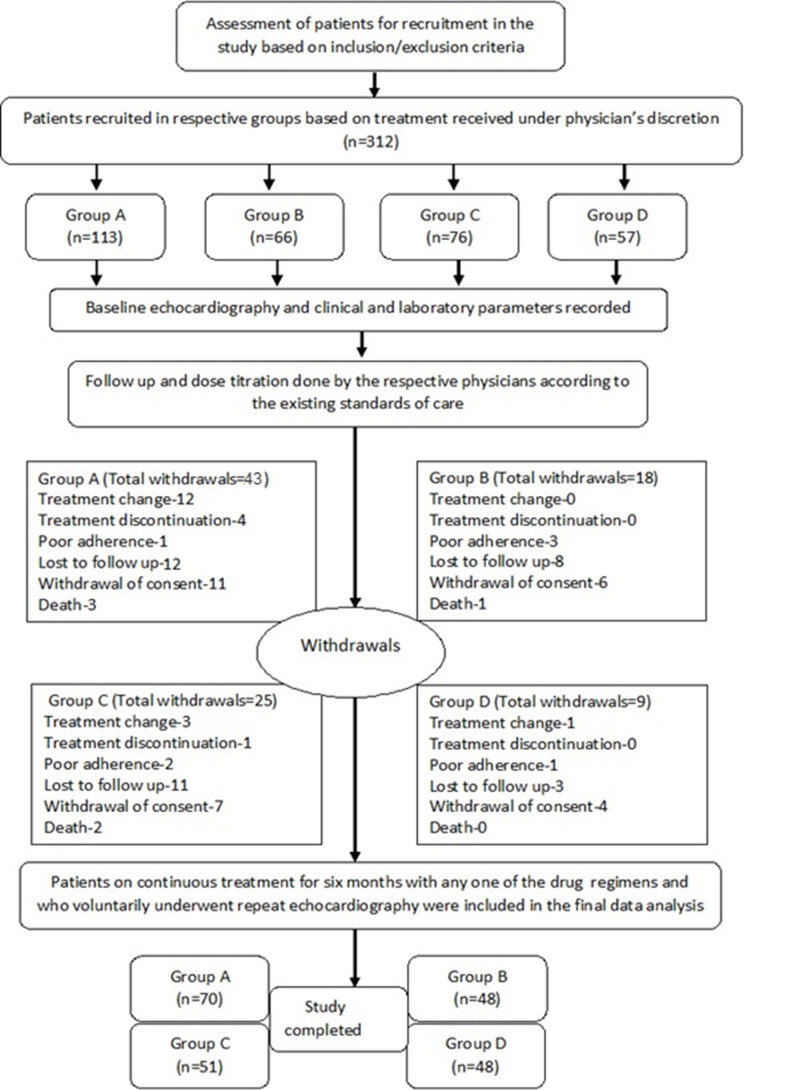
Flow-diagram of the study protocol A total of 312 patients were recruited in the study under four treatment arms. 95 patients could not complete the study for different reasons. The data of 217 patients was available for final analysis.

A total of 312 patients were recruited in the study based on eligibility criteria. All participants provided written informed consent. The data of 217 patients was available for final analysis. Subjects were excluded from final analysis on the basis of lost to follow up, treatment discontinuation, change of treatment, poor adherence, withdrawal of consent or death (Figure 2).

### Baseline patient characteristics: drug therapy

All the patients were on ACEI/ARB based regimen. Monotherapy consisted only of ACEI/ARB (group A). Dual therapy comprised of combination of ACEI/ARB with either a TD (group B) or a CCB (group C). Triple therapy consisted of all the three drugs (group D) (Table 1).

**Table 1:** Frequency distribution of different drug combinations in the four groups CTD-Chlorthalidone, HCTZ-Hydrochlorothiazide.

Group A (n=70)	Group B (n=48)	Group C (n=51)	Group D (n=48)
Enalapril (n=4)	Telmisartan+ HCTZ (n=30)	Telmisartan + Amlodipine (n=33)	Telmisartan+ Amlodipine + HCTZ (n=25)
Ramipril (n=3)	Telmisartan + CTD (n=7)	Telmisartan + Cilnidipine (n=8)	Olmesartan + Amlodipine + HCTZ (n=8)
Perindopril (n=2)	Olmesartan + HCTZ (n=6)	Losartan + Amlodipine (n=7)	Olmesartan+ Cilnidipine+ CTD (n=4)
Telmisartan (n=42)	Ramipril + HCTZ (n=3)	Olmesartan + Amlodipine (n=1)	Telmisartan +Amlodipine + CTD (n=3)
Losartan (n=13)	Olmesartan + CTD (n=1)	Azilsartan + Amlodipine (n=1)	Telmisartan + Cilnidipine + CTD (n=3)
Olmesartan (n=5)	Telmisartan+Ramipril + CTD (n=1)	Perindopril + Amlodipine (n=1)	Telmisartan + Cilnidipine + HCTZ (n=2)
Azilsartan (n=1)			Olmesartan + Amlodipine+ CTD (n=1)
			Losartan + Amlodipine + HCTZ (n=1)
			Enalapril + Amlodipine + HCTZ (n=1)

### Baseline patient characteristics: demographic and clinical

The comparative summary statistics of the baseline characteristics are shown in Table 2 and Table 3. There were no significant differences between the groups with respect to age, weight, height, body surface area and pulse rate (Table 2). However, baseline blood pressure differed significantly between the treatment categories. Results of post hoc analysis using Bonferroni test showed that group D had higher blood pressure than group A (mean difference of 18, 5, 9 mm of Hg in systolic, diastolic and mean BP respectively, P<0.001). Duration of hypertension was also significantly higher in group D compared to group A (mean difference 4.01 years, P<0.001). No difference between groups was found with respect to the categorical variables listed in Table 3. Patients in group B and group C were on two drug therapy for hypertension. There was no significant difference between the two groups with respect to baseline demographic and clinical characteristics.

**Table 2:** Patient characteristics at baseline (continuous variables) The baseline characteristics of the continuous variables in the four groups. There was no significant difference between the four groups with respect to age, weight, body surface area and pulse rate. There was significant difference between the four groups with respect to systolic, diastolic and mean blood pressure, and duration of hypertension. All data are expressed as mean ± SD; BP-Blood pressure.

Variable	Group A n=70	Group B n=48	Group C n=51	Group D n=48	P value
Age (in years)	55.97±10.51	57.65±11.99	55.92±12.38	56.90±12.09	0.852
Weight (in Kgs)	70.28±13.22	72.18±11.21	69.41±14.58	69.72±12.51	0.721
Body surface area (in m2)	1.74±0.17	1.75±0.162	1.73±0.19	1.72±0.17	0.858
Pulse rate (per minute)	81±13	82±11	78±12	82±14	0.370
Systolic BP (mm of Hg)	153±13	160±17	162±16	171±23	0.000
Diastolic BP (mm of Hg)	91±9	94±10	90±13	97±13	0.015
Mean BP (mm of Hg)	112±8	116±10	114±11	121±14	0.000
Duration of hypertension (in years)	3.94±3.5	7.47±6.7	5.41±5.3	7.95±6.8	0.000

**Table 3:** Patient characteristics at baseline (categorical variables) Baseline characteristics of the categorical variables in the four groups. There was no significant difference between the four groups with respect to the categorical variables. All Data expressed as counts; IHD- Ischemic heart disease, H/O- History of.

Variable	Group A n=70	Group B n=48	Group C n=51	Group D n=48	P value
Gender (Women/men)	24/46	19/29	20/31	20/28	0.859
H/O IHD (no/yes)	52/18	32/16	42/9	35/13	0.357
H/O Diabetes (no/yes)	44/26	26/22	32/19	37/11	0.129
Dyslipidaemia (no/yes)	62/8	41/7	44/7	42/6	0.961
H/O previous medication (no/yes)	15/55	7/41	11/40	6/42	0.500

### Baseline patient characteristics: echocardio-graphic parameters

The baseline echocardiographic parameters are shown in Table 4. There were no significant differences between the groups with respect to left ventricular dimensions and ejection fraction. However, significant differences were observed in LVMI between group A and group D (A < D, P=0.023).

**Table 4:** Patient characteristics at baseline: echocardiography Baseline characteristics of the echocardiography parameters in the four groups. There was no significant difference between the four groups with respect LVID, IVST, PWT, EF and RWT. There was significant difference between the four groups with respect to LVM and LVMI. All data are expressed as mean ± SD; LVID-Left ventricular internal diameter, IVST-Interventricular septal thickness, PWT-Posterior wall thickness (Left ventricular dimensions are in diastole), EF-Ejection fraction, RWT-Relative wall thickness, LVM- Left ventricular mass, LVMI-Left ventricular mass index.

Parameters	Group A n=70	Group B n=48	Group C n=51	Group D n=48	P value
LVID	3.99±0.54	4.10±0.51	4.02±0.58	4.10±0.62	0.85
IVST	1.30±0.15	1.32±0.16	1.28±0.13	1.35±0.25	0.72
PWT	1.27±0.16	1.30±0.16	1.27±0.14	1.35±0.26	0.86
EF	65.10±4.25	65.79±3.92	64.82±4.65	65.74±5.52	0.37
RWT	0.65±0.15	0.64±0.13	0.65±0.13	0.67±0.17	0.91
LVM	185±43	199±46	186±49	209±66	0.05
LVMI	107±23	114±26	108±27	122±36	0.02

### Efficacy analysis

#### Primary outcome (change in LVMI)

**Figure 3:**
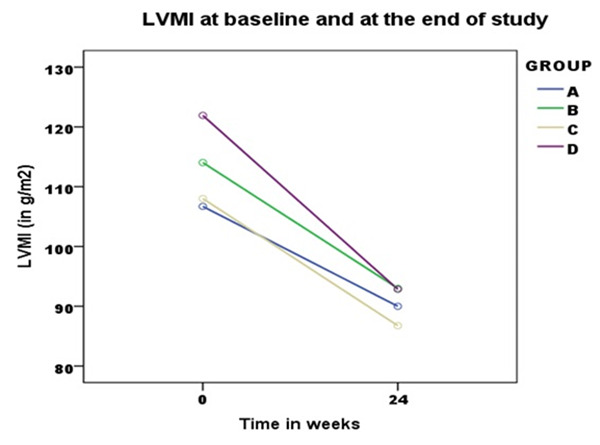
Time course of the change in LVMI in the four groups The mean baseline LVMI and mean LVMI at the end of 24 weeks of drug therapy are shown in the graph.

There was significant decrease in LVMI in all the four treatment arms after 6 months of treatment (P <0.001). The minimum and maximum reduction in LVMI was in group A (16.70±18.67) and Group D (29.08±21.49) respectively. Groups B and C have almost equal reduction in LVMI (21.03±20.79 and 20.48±15.50 respectively) (Figure 3).

A one-way ANOVA to detect treatment differences amongst groups was applied. The F value was found to be statistically significant F(3, 213)=3.263, P=0.022). Post hoc Tukey’s HSD test showed a mean difference of 12.38 gm/m^2^ between group D and group A (SE=3.97, P=0.011). Notably, no significant difference was observed between group B and group C. Pairwise comparison of other groups was not found to be statistically or clinically significant. Since the ANOVA test does not account for the covariates and the baseline differences, the validity of the test results is applicable to mutually exclusive treatment groups only. In such classification design, treatment effects are not freely transferable across the study cohorts and it is not possible to eliminate the errors arising out of baseline differences. The proportion of error in this ANOVA model is high (95.6%) and the portion of variability in the response variable (change in LVMI) explained by the treatment factors is only 4.4%. Application of ANCOVA increases the explained variability by introducing covariates linearly correlated with the response variable in the statistical model. In our ANCOVA model, the proportion of error decreased to 61% when pre-test LVMI was added as a covariate. The total explained variability increased to 39%. No significant interaction between treatment arms and baseline LVMI was found (P=0.830 for group*LVMI interaction in ANCOVA model). Baseline systolic BP was found to be a significant correlate (P=0.02) for change in LVMI but was found to be statistically insignificant in multivariate regression and treatment interaction effect, and therefore excluded from the final ANCOVA model. However, use of ANCOVA in a non-randomised observational study increases the probability of a type I or type II error due to inhomogeneity of regression slopes or regressor independence respectively. We observed that the significant difference between group A and D become non-significant when ANCOVA was applied. This is due to type II error and therefore statistical results of between group differences in a classification design using ANCOVA model should be rejected. On the other hand, absence of significant baseline differences confers a quasi-experimental design for comparison between group B and group C that increases the power of ANCOVA test. Although, there was an increase in the mean difference between the means of dependent variable between group B and group C in the ANCOVA test as compared to ANOVA (2.98 versus 0.22), the difference between groups B and C continued to be statistically non-significant.

#### Secondary outcome (change in BP)

**Figure 4:**
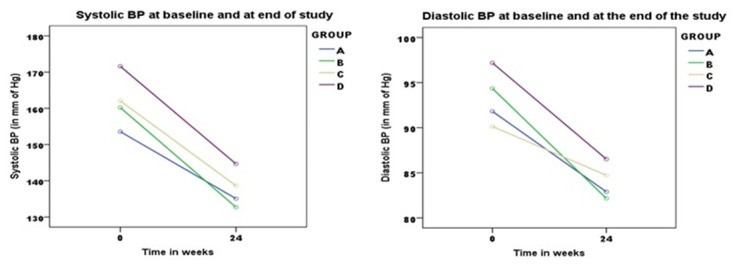
Time course of change in systolic BP (SBP) and diastolic BP (DBP) in the four groups The mean SBP and DBP at baseline and at the end of 24 weeks of drug therapy are shown in the graph. There was significant decrease in SBP and DBP in all the four groups (P<0.001).

Significant reduction in blood pressure was found in all four treatment groups at the end of the study period. Maximum decrease in systolic (27.50±19.23) and diastolic BP (12.19±9.284) was seen in group B. The minimum decrease in systolic (18.47± 13.75) and diastolic BP (5.41±10.145) was seen in group A and group C respectively (Figure 4). A one-way ANOVA was conducted to detect between group differences in reduction of blood pressure. Significant difference in reduction of systolic BP was found between group B and A (mean difference=9.04 mm of Hg, P=0.027) and between group D and group A (mean difference=8.34 mm of Hg, P=0.048). In case of diastolic BP, there was greater reduction with TD based therapy (group B) as compared to CCB based therapy (group C) (mean difference=6.8 mm of Hg, P=0.013).

## DISCUSSION

Hypertension is a leading cause of cardiovascular mortality and morbidity. The development of left ventricular hypertrophy (LVH) in hypertension is an independent predictor of adverse cardiovascular outcomes. Conversely, regression of LVH is associated with better prognosis. Lowering of blood pressure in patients with hypertension is the unequivocal determinant of regression of LVH^23,24^. For the same reduction in BP, it is seen in previous studies that the decrease in LV mass is maximum with ACEI/ARB in comparison to the other first line drugs when used as monotherapy^12-15^. However, evidence in contemporary literature comparing efficacy of ACEI/ARB in combination with other anti-hypertensives in regression of LVH is inconclusive. This necessitates the conduct of further studies. The present work is an observational prospective study where comparison between four naturally occurring cohorts based on ACEI/ARB regimens was carried out. The study also involved a direct comparison of efficacy between ACEI/ARB + TD and ACEI/ARB + CCB. In this report, we have discussed the findings of our study in light of recent literature and current practice guidelines.

### Comparison Between TD and CCB as Add-on Therapy

ACEIs/ARBs, unless otherwise contraindicated, are drugs of first choice among the first line antihypertensive drugs in hypertensive heart disease. A majority of patients require an add-on drug in the long run for tight control of blood pressure. The add-on drug is either a TD or a CCB, which in absence of a specific indication, is non-judgemental^17^.

#### Results of our quasi-experimental study

In our study, we compared the efficacy of TD (group B) and CCB (group C) in patients who were receiving this drug as a part of combination regimen based on ACEIs/ARBs. Systolic BP reduction in both the groups was similar whereas the diastolic BP reduction was significantly greater in the TD group (mean difference=6.8 mm of Hg, P=0.013). No significant difference in LV mass regression was found between the two groups at the end of the study period. The mean difference in the ANCOVA model between the two groups (group C > group B) was 2.98 g/m^2^ (P=1.000, CI=-6 to 12). Based on our result, we infer that add-on therapy in patients with essential hypertension not controlled with monotherapy of ACE inhibitor/ARB can be initiated with either a TD or a CCB without significant difference in regression of LVH.

*Results of previous studies and* *choice of add-on therapy*

The results of similar studies done in the past depict a variable picture. The Ehime-Study of Effective Anti-hypertensive therapy on Regression of Cardiac Hypertrophy (E-SEARCH), which compared losartan with hydrochlorothiazide (HCTZ) (losartan/HCTZ group) versus ARB plus CCB (ARB + CCB group) was a prospective, randomized clinical trial involving 53 patients. Systolic and diastolic BP decreased significantly by the same degree after 4 weeks in the two treatment groups. Losartan/HCTZ produced greater reduction in LVMI (137.3 ± 33.6 g/m2 to 114.9 ±33.4 g/m2, P=0 .016; mean change in LVMI –21.9 ± 38.3) compared to ARB +CCB (145.8 ± 44.0 g/m2 to 133.6 ± 43.7 g/m2, P =0.146; mean change in LVMI =11.3 ±33.3). The decrease in LV mass correlated with the decrease in inter-ventricular septal thickness and posterior wall thickness^25^. Conversely, a study by Boydak et al. in 2004 found that lisinopril + nifedipine was superior to lisinopril + hydrochlorothiazide in decreasing cardiac enlargement at the end of 6 months of treatment^26^. In the ALIVE (The Assessment of Lotrel in Left Ventricular Hypertrophy and Hypertension) study, CCB/ACEI (amlodipine/benazepril) versus a diuretic/ACEI (hydrochlorothiazide/benazepril), a greater reduction of 3.5gm/m2 was observed with amlodipine group but it was not statistically significant^27^. Out of the four trials which compared TD versus CCB in ACEI/ARB based regimen, one each favoured TD and CCB and the other two could not find any statistically significant difference between the two groups. Our study also shows that TD and CCB are equally efficacious in regression of LVH in hypertension patients. In clinical practise, the selection of second drug is generally decided on the basis of underlying comorbid conditions.

### Comparison Between Monotherapy, Dual Therapy and Triple Drug Therapy

#### Changing guidelines vis-à-vis our study

Early detection, prompt initiation of therapy and adherence to treatment are important goals of management of hypertension essential for achieving long term improvement in mortality and morbidity. The recommendations of Joint National Committee on prevention, detection, evaluation, and treatment of high blood pressure, which was in force till 2017 advocated the use of a single first line drug for stage I hypertension (140-159/90-99 mm of Hg). If target BP of ≤140/90 was not attained, then drug dosages were optimized, or additional drugs are added^28^. The ensuing guidelines in 2017 from the ACC/AHA was a paradigm shift in the approach to treatment of essential hypertension. It not only changed the staging of hypertension but also overhauled the blood pressure targets and treatment strategy. The committee recommended initiating antihypertensive drug therapy in stage 1 (130-139/80-89 mm of Hg) and stage 2 hypertension (≥140/90 mm of Hg) with monotherapy and combination therapy respectively. After initiation of antihypertensive treatment, regardless of the atherosclerotic cardiovascular risk, the recommended BP target is less than 130/80 mm Hg^29^. The following year (in 2018), the European Society of Hypertension came up with its own guidelines with a different staging and treatment recommendations. Although the recommendation was still focused on combination therapy from the beginning of treatment, it considered that a single drug is suitable for low-risk patients with stage 1 hypertension whose SBP is <150 mmHg^17^. At the offset, guidelines for treatment with monotherapy or combination therapy remained obscure. Our observational study was conducted during this transition period, when new and changing guidelines were broadcast in quick succession. The protocol of the study was planned and conceived in the “JNC7/8 era”, its inception coincided with release of ACC/AHA 2017 guidelines and recruitment continued after ESC/ESH 2018 guidelines were published. By the time the study was completed, we had the opportunity to recruit the required number of subjects into four naturally occurring cohorts (based on treatment regimens) and compare the treatment effects with the prespecified statistical power.

#### Results of our classification design study

A one-way ANOVA was applied, which showed that there was significant difference between the four groups in terms of regression of LVMI, as well as reduction of BP. In our discussion in this paragraph, we will focus on differences between monotherapy and combination therapy as they are relevant in light of the recently changed guidelines. We observed a trend that the decrease in BP and LVMI increased progressively as therapy was stepped up, indicating that combination therapy is superior to monotherapy. The maximum reduction in LVMI was observed with three drug regimens (group D=ACEI/ARB+ TD+ CCB). The reduction in LVMI in group A, group B, group C and group D was 16.70, 21.03, 21.26 and 29.08 g/m^2 ^respectively. The corresponding systolic and diastolic BP reductions were 18.47, 27.50, 23.39, 26.63 and 8.91, 12.19, 5.41, 10.67 mm of Hg respectively. Paradoxically, the maximum reduction in systolic and diastolic BP was seen in group B while the maximum regression of LVMI was in group D. Since group D was a combination of all the three drugs, it can be hypothesized that the individual class of drugs have preventive cardiac remodelling effects over and above the reduction of blood pressure. The collective action of all the three drugs induced the maximum regression of LV mass in group D.

#### Comparison with previous studies

In reports of previous literature, the same trend has been documented. In the PICXEL study, combination of perindopril with indapamide was found superior to enalapril alone in regression of LVMI and reduction of blood pressure^30^. It was assumed that the addition of the diuretic produced a synergistic effect on regression of LVMI, by decreasing volume overload on the left ventricle. It significantly reduced left ventricular internal diameter (LVID) in addition to the interventricular septal thickness (IVST) and posterior wall thickness (PWT). Enalapril monotherapy, on the other hand, reduced IVST only. The results of our study are similar to PICXEL study in terms of reduction of LV mass and BP. However, they differed with respect to the change in ventricular dimensions. In our study, there was no significant difference in the decrease in LVID between the groups. However, IVST and PWT decreased significantly in all the groups. There was greater reduction in IVST and PWT in diuretic group compared to monotherapy which, however, was not statistically significant. The reduction in LV dimensions may be both pressure and volume dependent, because we found that combination of CCB with ACEI/ARB was equally efficacious with the combination of TD with ACEI/ARB. We found that the decrease in BP and the baseline LVMI were more important determinants of regression of LVMI than the drug itself. The authors of the PICXEL study had also pointed out that greater regression in LVMI may be due to greater BP reduction, but they did not take into account the baseline LVMI. Monotherapy had also been compared with CCB based combination therapy by some researchers. Neutel et al. conducted a randomised controlled trial involving 106 patients distributed into three groups, aged 18 years or more with mild-to moderate hypertension. They found that there was a significant decrease in BP and LVM in all the three groups. It was observed that combination therapy of benzapril + amlodipine (n=35) induced greater regression than benzapril (n=34) or amlodipine (n=37) alone. The difference with amlodipine group reached statistical significance^31^. In both these studies, as well as our study, combination therapy produced greater reduction of BP and LVMI than monotherapy.

#### Debate on intensive versus standard lowering of BP

Two landmark studies, Action to Control Cardiovascular Risk in Diabetes–Blood Pressure trial (ACCORD, n=4733) and The Systolic Blood Pressure Intervention Trial (SPRINT, n=8164) compared the effect of intensive BP lowering against standard BP lowering in hypertensives with diabetes and without diabetes respectively. Both studies showed that there was greater regression of LVH and lesser development of new LVH with intensive BP lowering^11,32^. The SPRINT trial also observed that the cardiovascular benefit obtained with intensive lowering of BP could not be attributed solely to the magnitude of LVH regression. Intensive BP reduction imparted additional cardiovascular protection over and above obtained with prevention of cardiac remodelling^32^. In recent guidelines, a prompt shift to combination therapy is recommended if the target BP is not achieved in the specified period. There is an unmet need to confirm the newly discovered evidence on intensive BP lowering with further studies. Since BP reduction is dose dependent, there is a scientific possibility of comparing combination therapy with monotherapy maintaining the highest ethical standards^33^. In the SPRINT trial, diuretic use in the intensive and standard treatment arm was 67% and 43% respectively^34^. Indirectly, this indicates that the proportion of change in LV mass that can be attributed to either drug or BP reduction are inter-related and inseparable, because BP reduction itself varies as a function of the drug class and dose. In other words, both BP reduction and drug class may have independent roles in decreasing the rate of cardiovascular events^35^. This further justifies the beneficial effect of using combination therapy in the treatment of hypertension^34^. In our observational study, the study groups were mutually exclusive, non-randomized cohorts under treatment according to the existing standards of care. Though treatment effects are not transferable across groups in such a study design, the results reinforce the recommendation of existing guidelines that triple drug therapy should be used to achieve the target blood pressure whenever indicated. Despite the unexplained part, it is evident that these studies, as well as our own study, show that combination therapy is superior to monotherapy in achieving the desired objectives.

## LIMITATIONS

Our study was a prospective, observational cohort study utilising non-probability sampling. Randomisation and blinding were not done. Although systematic reviews and meta-analysis have shown that there is no significant difference in effect size of treatment between observational and randomised studies, there is a high chance of observer bias when blinding is not implemented^36-38^. We used office BP as the secondary outcome for efficacy analysis in our study. We measured office BP using a regularly calibrated aneroid BP instrument in a patient, rested and seated properly for 5 minutes. The mean of two readings taken 2 minutes apart was recorded as the office BP. Office BP readings reflect only an instantaneous picture of a patient’s true BP and provide no idea about the diurnal variation in BP or the variation in BP due to the drug itself. Recent research has shown that ambulatory blood pressure is better than office BP in diagnosis and monitoring of hypertension and can be a useful predictor of cardiovascular risks^39^. Use of ambulatory blood pressure in our study would have provided more realistic results but could not be done due to limitation of cost and resources.

Echocardiography is the standard clinical method for estimation of left ventricular mass. Though the inter-observer and intra-observer variability are not clinically significant, correlation between echocardiography determined LV mass and post-mortem measurement (gold standard) showed that echocardiography explains 85% of the variance^40^. Cardiac magnetic resonance imaging provides better resolution and quantification of LV mass but low cost, real time imaging and prompt interpretation makes echocardiography popular in clinical practise. In studies where regression of LV mass is the primary outcome, magnetic resonance imaging should be the preferred imaging modality^41^. The proportion of variation in treatment effect due to errors in measurement of LV mass in echocardiography method lies undetermined in our study. The benefits of antihypertensive treatment on cardiovascular morbidity and mortality is ascribed to the normalisation of LVH. Under this perception, the outcome variable of our study was the magnitude of change in ventricular mass. The reversal of LVH is considered to be the surrogate marker of the efficacy of antihypertensive therapy. However, the factual objective of treatment in hypertension is the reduction in the rate of adverse cardiovascular events (RACE). Though a direct relationship exists between regression of LVH and RACE, the differential role of specific drug class in reduction of RACE is an independent subject-matter, beyond the scope of our study^42^.

## CONCLUSIONS

Anti-hypertensive treatment with ACEI/ARB based monotherapy, dual therapy and triple therapy produces a graded decrease in LVH, matching with the magnitude of BP reduction. Our findings support the current recommendation of prompt shifting to combination therapy to achieve tighter BP control and hence greater cardiovascular benefit. In patients requiring dual therapy for BP control, add-on of either TD or CCB to ACEI/ARB have equal efficacy in regression of LVH independent of BP reduction. We, therefore, conclude that add-on therapy in patients with essential hypertension not controlled with monotherapy of ACEI/ARB can be initiated with either a thiazide diuretic or a calcium channel blocker without significant difference in regression of LVH.

**Take Home Messages**

In our observational cohort study, comparing regression of left ventricular hypertrophy (LVH) in patients of uncontrolled essential hypertension on various ACEI/ARB (angiotensin converting enzyme inhibitor/ Angiotensin receptor blocker) based regimens, the following are the key findings.

## KEY POINTS

· ACEI/ARB based antihypertensive regimens lead to significant decrease in blood pressure, as well as regression of LVH at the end of six months of treatment.

· There is no significant difference in regression of LVH between the two dual drug regimens (ACEI/ARB+ thiazide diuretic versus ACEI/ARB+ calcium channel blocker) at the end of the study. Thus, we conclude that add-on therapy in patients with essential hypertension not controlled on monotherapy with ACEI/ARB can be initiated with either a thiazide diuretic (TD) or a calcium channel blocker (CCB) without significant difference in regression of LVH.

· Both systolic and diastolic blood pressure are significantly reduced in all the four groups after six months of treatment. Maximum reduction in systolic, as well as diastolic blood pressure, occurs in patients receiving ACEI/ARB + TD but maximum reduction in LV mass is seen in those on combination of ACEI/ARB+ TD+ CCB.

· Greater reduction in both systolic (statistically non-significant) and diastolic blood pressure (statistically significant) occurs when a diuretic is added to ACEI/ARB compared to addition of CCB. However, greater regression of LVH (statistically non-significant) occurs with ACEI/ARB+ CCB.

· Combination therapy induces greater regression of LV mass and clinicians should be encouraged to promptly initiate add-on therapy whenever indicated as per the latest guidelines.

· Baseline left ventricular mass index (LVMI) and change in systolic blood pressure are most important non-pharmacological determinants of regression of left ventricular hypertrophy. Higher baseline LVMI and greater reduction in systolic blood pressure are associated with greater regression of LVMI.
